# How can we cure a heart “in flame”? A translational view on inflammation in heart failure

**DOI:** 10.1007/s00395-013-0356-y

**Published:** 2013-06-06

**Authors:** Ulrich Hofmann, Stefan Frantz

**Affiliations:** 1 Department of Internal Medicine I, University Hospital Würzburg, Oberdürrbacher Straße 6, 97080 Würzburg, Germany; 2 Comprehensive Heart Failure Center, University of Würzburg, Straubmühlweg 2a, Würzburg, Germany

**Keywords:** Cytokines, Heart failure, Immuno-modulation

## Abstract

The prevalence of chronic heart failure is still increasing making it a major health issue in the 21st century. Tremendous evidence has emerged over the past decades that heart failure is associated with a wide array of mechanisms subsumed under the term “inflammation”. Based on the great success of immuno-suppressive treatments in auto-immunity and transplantation, clinical trials were launched targeting inflammatory mediators in patients with chronic heart failure. However, they widely lacked positive outcomes. The failure of the initial study program directed against tumor necrosis factor-α led to the search for alternative therapeutic targets involving a broader spectrum of mechanisms besides cytokines. We here provide an overview of the current knowledge on immune activation in chronic heart failure of different etiologies, summarize clinical studies in the field, address unresolved key questions, and highlight some promising novel therapeutic targets for clinical trials from a translational basic science and clinical perspective.

## Introduction

Congestive heart failure (CHF) is a leading cause for both hospitalization and death in the western world. Its prevalence is rather increasing with the broad implementation of standardized evidence-based treatment algorithms for heart failure. The heart failure syndrome is characterized by impaired systolic and/or diastolic function and various clinical signs such as fatigue, dyspnea, fluid retention, and cachexia. Besides symptomatic treatment to relieve these symptoms several classes of drugs improve the prognosis of patients with chronic heart failure. These agents target the so-called neurohumoral activation process that comes along with the evolution of the heart failure syndrome irrespective of the underlying pathophysiology. Inhibition of the activity of the renin–angiotensin–aldosterone system and β-adrenergic signaling are well-established treatment principles which are incorporated in current guidelines [100]. However, morbidity and mortality remain substantial in chronic heart failure patients despite optimal medical and device therapy, the indication for which has recently been broadened [100].

**Fig. 1:**
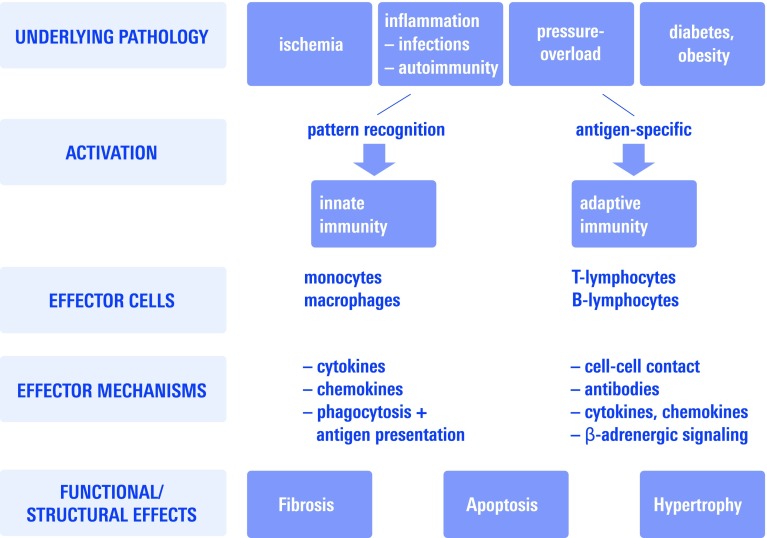
Schematic overview depicting activation mechanisms of both innate and adaptive immunity and their central effector mechanism reviewed here

An inflammatory activation in CHF patients has long been recognized. Indeed, immune mechanisms modulate interstitial fibrosis, cardiomyocyte apoptosis, and hypertrophy, all of which are central processes leading to maladaptive remodeling in response to a variety of stimuli (Fig. 1). Especially for heart failure evolving from large myocardial infarction there is substantial evidence for a causal contribution of immunity early in the course of the disease.

First, systemic cytokines came into focus, which have been monitored in several clinical trials [33, 112]. The broadest amount of data was gathered for tumor necrosis factor-α (TNF-α) [78] which was demonstrated to correlate well with diverse clinical and laboratory parameters, such as exercise capacity and neurohormonal activation in CHF patients [26]. These observations lead to the conduction of clinical trials testing anti-TNF-α drugs in chronic heart failure patients [25, 96]. Their discouraging results have raised important questions on the significance of the inflammatory activation in chronic heart failure. It is still unresolved whether these trials treated the right patients with the right drugs [4] or whether the whole concept of anti-cytokine therapy mainly based on small animal studies does not work in a heterogeneous population of patients with chronic heart failure of diverse etiology.

Meanwhile, basic science and translational clinical research gathered a lot of mechanistic information on new players in the field of inflammation which constitute very promising future targets for therapy. Therefore, a recent scientific statement from the European Society of Cardiology defined key questions and requirements for future studies in the enormously expanding field [61].

The current review will provide the reader with an elected overview of experimental and clinical evidence for immune activation in chronic heart failure, summarize key clinical trials addressing the immune system, and provide an outlook for several new promising clinical targets.

## Evidence for immune activation in heart failure of different etiology

Different etiologies like coronary artery disease, hypertension, infection, etc., lead to heart failure. Therefore, one of the key questions in the field is whether activation of immunity in humans and animal models of heart failure is independent of the etiology or if there are disease specific mechanisms. As it turns out, both are the case.

Different heart failure models indicate a role of innate immunity independent of heart failure etiology. Innate immunity is activated in the myocardium early by recognition of rather unspecific stimuli, summarized as danger-associated molecular patterns [10]. This form of sterile inflammation is prototypically initiated by engagement of innate pattern recognition receptors, like toll-like receptors [68].

It is likely that inflammation is also initiated in human myocardium by innate recognition of pathogen-associated molecular pattern even well before the heart failure becomes symptomatic/diagnosed. However, clinical data to corroborate findings made in animal studies are widely limited to the demonstration of increased circulating levels of soluble mediators, mainly cytokines [78], in a variety of patient cohorts with heart failure.

For heart failure with reduced ejection fraction due to different clinical etiologies there is good evidence from both experimental and clinical studies that pro-inflammatory cytokines are locally expressed in myocardium. For example, TNF-α has been mechanistically related to more or less all etiologies of systolic heart failure. Besides its direct effect on myocardial contractile function [35, 155], TNF-α contributes to progressive myocardial remodeling. This is underscored by the demonstration that chronic systemic administration of TNF-α in concentrations found in CHF patients [14] or its transgenic myocardial expression produces a DCM-like phenotype [79].

Nevertheless, there are also distinct differences in the activation of the immune system in different heart failure etiologies:

### Ischemic heart failure

Heart failure due to large myocardial infarction is the most prevalent cause of systolic heart failure, so the majority of small animal studies for heart failure were conducted in the permanent myocardial infarction model. Besides early infiltration of leukocytes and local expression of cytokines and chemokines, there is ongoing inflammation that extends to the remote myocardium and is a part of the underlying pathophysiology of left ventricular remodeling. A detailed review of all the processes involved in myocardial remodeling is beyond the scope of the present article and was summarized recently [42]. Inflammation mainly involves innate immunity mechanisms including toll-like receptor activation [44, 68] and NFκB intracellular signaling both in local cells (cardiomyocytes, fibroblasts) and in infiltrating leukocytes [45]. Clinical evidence for the activation of adaptive immunity is comparatively sparse. However, the increased prevalence of auto-antibodies against structural components of [30] and cell surface receptors on cardiomyocytes [129] indicates that ischemic injury can induce an adaptive auto-immune response against myocardial tissue.

Most animal studies are performed in relatively acute settings with follow-up of no longer than 8 weeks. Especially experiments with genetically altered mice often do not really allow distinguishing the effect on initial myocardial injury and wound healing, when the most pronounced inflammatory activation takes place, from chronic remodeling of the myocardium. Therefore, therapeutic approaches based on such animal experiments rather reflect the clinical situation weeks to months after a myocardial infarction when mechanisms, such as infarct expansion are still operative and do not easily allow transfer of the findings to the situation of chronic, non-ischemic, systolic heart failure.

In contrast to the broad evidence from small animal studies, due to the limited availability of myocardial specimens clinical evidence for immune activation in chronic ischemic heart failure mainly comes from analysis of cytokines and cytokine receptors in peripheral blood [8] which first led to the design of anti-cytokine trials, as summarized below.

### Inflammatory heart disease

The threshold of >14 leukocytes/mm² is widely accepted for the definition of myocardial inflammation [113]. Endomyocardial biopsy allows further differentiation between virus-positive and virus-negative inflammatory cardiomyopathy. Human herpesvirus 6 and Parvo B19 virus are the most prevalent viruses found in myocardial biopsy specimens from patients with suspected myocarditis [94]. Besides direct virus mediated cardiac injury, the ensuing immune response, mainly studied in coxsackievirus B3 small animal models of myocarditis, significantly contributes to myocardial injury and to the development of chronic heart failure [28]. Transition into sustained myocardial inflammation despite halted virus replication by induction of auto-immunity further propagates local inflammation and eventually leads to progressive myocardial fibrosis with transition to the clinical entity DCM [27]. Animal models of myocarditis demonstrated that the genetic background plays an important role for modulating the adaptive immune response, involving the cooperation of innate immune cells and lymphocytes for induction of persistent myocardial inflammation even after virus control as a transition to auto-immunity [36]. However, we still do not really understand why some patients control or even eliminate viral infection of the heart, whereas others develop progressive deterioration leading to DCM. This also relates to the unknown pathophysiological significance of a high prevalence of viral genome without evidence of local inflammation, even in healthy hearts [80, 91]. Referring to this, Kindermann et al. [76] reported recently that the detection of immunohistological evidence of inflammatory infiltrates and HLA class II expression in the myocardium rather than detection of virus genome is of prognostic relevance in patients with suspected myocarditis.

It is further unclear whether, in analogy to mouse models of auto-immune myocarditis induced by vaccination against myocardial proteins, auto-immune myocarditis develops in patients without previous infection of the myocardium. Data from the TIMIC trial [48] demonstrating that a differentiation into virus-positive and -negative inflammatory cardiomyopathy identifies patients which could benefit from immunosuppression by steroids + azathioprine indicate that auto-immune cardiomyopathy might indeed constitute a pathophysiologically distinct clinical entity. This does not exclude that myocardial auto-immunity in man might always be initiated by the adjuvant effect of concomitant myocardial or systemic infection which might break tolerance to myocardial structural components. Thus, at the moment, a careful analysis of the presence of virus genome in the myocardium in an experienced laboratory seems to be mandatory, at least for further clinical trials in the field.

### Heart failure with preserved ejection fraction, myocardial hypertrophy

Chronic pressure overload also leads to the expression of chemokines, cytokines, and infiltration of monocytes in the myocardium. Most evidence comes from animal models of transverse aortic constriction. There is a well-described pathophysiological link between inflammation and fibrosis [13, 83], whereas the link to hypertrophy is less well established. Especially macrophages play a central role for development of interstitial fibrosis in response to different stimuli. Alternatively activated macrophages induce fibrosis by expression of proteins like arginase, coagulation factor XIII, and TGF-β [151]. Experimentally, T-cells contribute to the pathophysiology of myocardial fibrosis. T-cell derived IL-18 can induce osteopontin expression and fibrosis in myocardium [157]. In a mouse model of hypertension, lymphocyte deficient SCID mice were protected against myocardial collagen accumulation. There was less fibrillar collagen cross-linking and reduced activity of the cross-linking enzyme lysyl-oxidase-like-3 compared to wild type C57B6 mice and even higher collagen content in Th2 biased BALB/c mice [156] indicating that CD4^+^ T-cells, especially modulate collagen deposition in myocardium.

An expression of pro-inflammatory cytokines was also described in myocardium from patients with aortic stenosis [146]. Here, the pathomechanism underlying the transition from adaptive hypertrophy to irreversible remodeling and decompensation is incompletely understood and might also implicate fundamental changes in the inflammatory response.

Inflammation in heart failure with preserved ejection fraction without overt hypertrophy has been less well characterized. In patients with coronary artery disease and diabetes, a small study found an association between pro-inflammatory serum cytokines and echocardiographic evidence of diastolic dysfunction [34].

In sum, clinical evidence for the implication of inflammation in the development of myocardial hypertrophy and diastolic dysfunction is sparse, although experimental studies suggest a role especially for lymphocytes and macrophages.

### Diabetes and obesity

More and more evidence is accumulating that obesity is associated with a systemic inflammatory response that is characterized by endothelial cell dysfunction, oxidative stress, and the activation of leukocytes [118]. The inflammatory response mediated by hormones from adipose tissue, like adiponectin, might contribute to cardiac remodeling in obesity [109]. Data on the functional status of the myocardium in advanced obesity are ambiguous reporting either normal or impaired diastolic, some even impaired systolic function in different cohorts [2]. It is thus still not quite clear, whether obesity leads to heart failure independently from coronary heart disease.

The amount of epicardial fat was often correlated with the severity of hypertrophy and cardiac dysfunction as evidenced mainly by non-invasive imaging with its inherent limitations in obese humans. Especially the adipocytokine adiponectin, which is decreased in patients with obesity-linked diseases, prevents left ventricular hypertrophy. Studies in adiponectin deficient mice revealed increased afterload-induced hypertrophy due to impaired activation of the activated protein kinase AMP [117]. However, the results from a variety of animal models of diabetes and obesity are ambiguous in terms of cardiac function and the contribution of metabolic changes concerning substrate utilization and hyperglycemia on the one hand, and adipose tissue-derived inflammation on the other, for the myocardial phenotype observed [2, 37, 158].

## Therapeutic approaches in clinical trials

### Cytokine targeting

The best studied pro-inflammatory cytokine in the scenario of heart failure is TNF-α [78, 102]. Clinical studies have shown elevated serum levels of TNF-α in patients with decompensated CHF, especially in those with cachexia [107]. There is further good evidence from experimental studies that TNF-α has an immediate negative effect on contractile function [35, 155] and the economy of contraction [67]. TNF-α elicits immediate negative inotropic effects mainly via binding to TNF-receptor 1 (TNFR1). Its activation induces the formation of sphingosine which reduces calcium release from the sarcoplasmic reticulum [47, 138] and decreases β-adrenergic receptor coupling leading to diminished sarcoplasmic reticulum calcium pump activity [82]. Furthermore, TNF-α increases ROS formation by uncoupling mitochondrial respiration [18] which in turn promotes myocardial dysfunction by oxidation of contractile filaments [24]. The clinical relevance of these findings has not yet been tested by a clinical trial testing neutralization of TNF-α in patients with acute/decompensated heart failure.

Besides its immediate effects on the contractile performance of the myocardium, there is also good experimental evidence for a causal pathophysiological role of TNF-α for the progression of myocardial remodeling leading to chronic heart failure. Relevant mechanism demonstrated in experimental studies are induction of cardiomyocyte hypertrophy [154], induction of cardiomyocyte apoptosis [160], and progressive left ventricular dilation by inducing matrix-protease activity [89].

Based on these extensive animal data indicating a causal link between TNF-α and cardiac dysfunction in both the acute and chronic setting, clinical trials were launched which tested compounds initially developed for binding of TNF-α in rheumatic diseases. The compound etanercept consists of two human TNF-α receptor fragments linked to the Fc portion of immunoglobulin G. In a phase I study, etanercept single administration was well tolerated and improved ejection fraction in chronic heart failure patients [32]. Etanercept treatment over 3 months then showed improvement in clinical parameters and ejection fraction [15]. These pilot trials confirmed the expectations based on animal studies and led to the initiation of large multicenter trials (RECOVER, RENAISSANCE, RENEWAL) testing the long-term effect of etanercept in chronic heart failure patients with NYHA III–IV functional status and EF ≤30 %. In fact, on the basis of pre-specified stopping rules, the trials were terminated prematurely. Etanercept had no effect on clinical status in RENAISSANCE or RECOVER and had no effect on the death or chronic heart failure hospitalization endpoint in RENEWAL, which analyzed the outcome from a high dose subgroup of patients of both trials [96].

Infliximab is a chimeric antibody binding soluble and membrane-bound TNF-α and prevents it from binding to its receptors. In a smaller phase II trial (ATTACH), NYHA III–IV patients treated with lower doses of infliximab demonstrated an increase in ejection fraction, but there was an increased risk of hospitalization and death in a high dose group [25].

Besides dosing and timing issues, two main points have been worked out as possible explanations for the disillusioning results [4]. First, cardiomyocytes can express TNF-α on the cell membrane [35, 142, 143]. Binding of infliximab is expected to induce apoptosis in cells expressing membrane-bound TNF-α. Second, etanercept and infliximab were suspected to prolong the half-life/bioavailability of TNF-α. Accordingly, patients treated with infliximab revealed increased concentrations of TNF-α [25]. This might explain why single doses of etanercept in the phase I study appeared to be beneficial, whereas long-term treatment is obviously detrimental.

The disappointing results of the anti-TNF-α studies in CHF patients dampened the enthusiasm in the field and supported a common belief that the redundancy of the immune system precludes success of specific cytokine-targeting strategies. Thus, although other pro-inflammatory cytokines were demonstrated to be involved in cardiac injury and significantly correlated with clinical status and prognosis of patients with chronic heart failure [56], especially IL-6 has to be mentioned here [75], there was no further prospective randomized clinical trial targeting cytokines in chronic heart failure patients. Several drugs approved for unrelated indications possess anti-inflammatory, anti-oxidant, or immuno-modulatory properties and were tested in patients with acute or chronic heart failure thereafter.

### Glucocorticoids

One of the first randomized trials on immuno-modulation was conducted years before the event of the anti-TNF-α trials. Glucosteroids were tested in a number of small studies before the advent of current reperfusion therapy. A number of rather small studies produced controversial results when patients were treated in the setting of an acute myocardial infarction to limit infarct size and improve wound healing [88]. Later on, glucosteroids were tested in CHF patients. In idiopathic DCM patients, prednisone treatment induced a very modest, transient effect on ejection fraction in a subgroup with histological evidence of inflammation [108]. Accordingly, combined treatment with glucosteroids and azathioprine showed a beneficial on left ventricular function in DCM patients selected by myocardial HLA up-regulation in myocardial biopsy specimens [150]. The utility of immunosuppression by steroids was proven later in the randomized, placebo-controlled TIMIC trial [48]. Patients with chronic biopsy proven virus-negative inflammatory cardiomyopathy benefited from prednisone/azathioprine treatment in terms of a significant improvement of left ventricular ejection fraction compared with both baseline and the control group. Yet, no long-term follow-up data with further clinical endpoints are available from this trial.

### Thalidomide

Thalidomide was initially believed to act as an anti-TNF-α agent. In a small pilot uncontrolled trial, thalidomide rather increased plasma levels of TNF-α in patients with chronic heart failure, but improved left ventricular function [54]. Thalidomide treatment for 12 weeks induced reverse remodeling of the left ventricle in a subsequent small randomized trial. Improvement in LVEF was accompanied by a decrease in matrix metalloproteinase-2 suggesting a matrix-stabilizing rather than an anti-inflammatory effect [55].

### Statins

Statins are well known for their “pleiotropic” effects besides cholesterol lowering. Immuno-modulatory and anti-inflammatory effects have been described which in part, inter alia, relate to attenuation of T-cell activation by repression of MHC-II expression [85]. Rosuvastatin was, therefore, studied in two clinical trials in patients with chronic heart failure. The CORONA trial tested the efficacy of rosuvastatin in a mixed CHF population [77]. Rosuvastatin did not reduce the primary outcome, although the drug did reduce the number of cardiovascular hospitalizations. In the unrelated GISSI-HF trial, rosuvastatin was also tested in patients with chronic heart failure, irrespective of underlying cause and left ventricular ejection fraction. Here, the drug did not affect pre-specified clinical outcomes [134]. Therefore, based on a fairly large cohort of chronic heart failure patients studied in clinical trials, rosuvastatin treatment could not prove a benefit as adjunct to current heart failure medication.

### Pentoxifylline

Pentoxifylline is a phosphodiesterase inhibitor which is approved for the treatment of peripheral vascular disease. The initial rationale for using pentoxifylline was due to its ability to inhibit TNF-α transcription. Yet, not all studies could link improvements in clinical outcome to a concordant effect on TNF-α modulation [116].

In several clinical trials, pentoxifylline therapy was reported to reduce circulating levels of C-reactive protein, FASL, and TNF-α in heart failure patients. In those small heart failure trials, pentoxifylline also improved ejection fraction and functional outcome [119, 120, 122, 124]. However, a clinical trial from an unrelated group demonstrated that treatment with pentoxifylline had no significant effect on left ventricular function, inflammatory cytokines, and symptoms in patients with cardiomyopathy of various etiologies [9]. Pentoxifylline was also tested in women with peripartum cardiomyopathy and produced promising effects on a combined outcome endpoint [121]. Whereas most studies included patients with mild-to-moderate heart failure, another trial tested pentoxyphilline in a small NYHA IV patient cohort. This prospective, placebo-controlled study revealed an increase in ejection fraction after 1 month [123].

### Anti-oxidants

Oxidative stress is believed to play a causative role in the evolution of the heart failure syndrome mainly on the basis of experimental studies. On the cellular level, reactive oxygen species can activate multiple signaling pathways, including MAP kinases and nuclear factor-κB [69]. This induces expression of pro-inflammatory cytokines like TNF-α, which again promotes ROS formation and pro-apoptotic signaling as a potential positive feedback mechanism after myocardial infarction or chronic pressure overload [153]. Myofibrillar protein oxidation was shown in an animal model of coronary microembolisation [24] and a pacing-induced heart failure model [60]. Of note, oxidative modification of myofibrillar proteins was subsequently also found in human failing hearts [23, 59]. A potential role for generation of oxidative stress was further underscored by the clinical observation that there is a significant positive correlation between the clinical class of heart failure and several biochemical parameters of oxidative stress in patients with chronic heart failure [73]. Therefore, a randomized trial was conducted to study whether oxypurinol, a xanthine oxidase inhibitor expected to reduce the production of reactive oxidant species, produces clinical benefits in patients with NYHA class III/IV heart failure. Oxypurinol did not produce clinical improvements [57]. The effect of allopurinol on diastolic function in chronic heart failure patients was further studied in a recent, yet unpublished, trial (NCT00477789).

Another small double-blinded controlled randomized trial aimed to determine whether vitamin E supplementation of patients with advanced heart failure (NYHA III/IV) would modify levels of oxidative stress and improve functional status and prognosis. However, supplementation with vitamin E did not result in any significant improvements [74].

Despite the theoretical potential of adding anti-oxidants to standard heart failure medication, which might have been underestimated on the basis of these rather small trials, it has to be mentioned that standard heart failure medication already includes drugs with anti-oxidant properties, especially ACE inhibitors and angiotensin receptor blockers [140].

On the other hand, the addition of a fixed dose of isosorbide dinitrate plus hydralazine to standard therapy for heart failure improved survival among black patients with advanced heart failure in the AHeFT trial [135, 136]. This drug combination includes a nitric oxide donor (isosorbide dinitrate) and an antioxidant (hydralazine) to protect NO from degradation. Anti-oxidant mechanisms improving NO bioavailability and, thus, endothelial dysfunction were discussed to contribute to the observed effect [40]. The results of AHeFT indicate that there might be considerable variation in the significance of the role of oxidative stress and endothelial function according to ethnical background.

### Immune modulation by modified autologous blood

Autologous blood exposed ex vivo to heat and oxidative stress induces anti-inflammatory effects in animal models [106]. The precise mechanism has not been studied, especially in humans. One might speculate that apoptotic cells contained in the autologous blood could have an important contribution to the immuno-modulatory effect [147]. Based on these experimental results, a small randomized phase II study was conducted in NYHA III–IV heart failure patients. This study demonstrated a significantly reduced risk of death and hospitalization [144]. The subsequent placebo-controlled ACCLAIM trial did not find a significant reduction of hospitalization or mortality in over 2,400 NYHA III–IV patients studied during 12 months [141].

### PUFA

Considerable epidemiological data found an association of omega-3 polyunsaturated fatty acid (ω-3 PUFA) intake, mainly from fish oils, and cardiovascular endpoints (reviewed in [87]). Reduction of coronary events seems to contribute most to the total effect. A recent prospective study of nearly 60,000 Japanese people with a high intake of fish, followed-up for over 12 years, revealed an inverse association between fish, PUFA consumption, and mortality, especially for heart failure [152]. PUFAs have multiple immuno-modulating properties including effects on arachidonic acid metabolism and peroxisome proliferator-activated receptors (PPARs) [103].

Dietary PUFA supplementation was studied by the Gruppo Italiano per lo Studio della Sopravvivenza nell’Infarto Miocardico-Heart Failure (GISSI-HF) study, a large-scale clinical trial in a chronic heart failure cohort. This trial showed a moderate, albeit significant benefit of ω-3 PUFA in addition to standard heart failure medication [133]. The mechanism behind it is not yet clear. The beneficial effect on mortality seemed not to rely on prevention of sudden cardiac death as in the GISSI prevention trial [1]. Recently, in a small double-blind, randomized, controlled trial two doses of PUFA were tested in patients with advanced, non-ischemic heart failure. Treatment with PUFA for 3 months led to a dose-dependent increase of LVEF [104].

Based on these trials the clinical relevance of PUFAs in chronic heart failure patients is still not clear. Thus, PUFA supplementation received a class IIb, level of evidence B recommendation in the most current European heart failure guideline [99].

### Immuno-adsorption, antibody neutralization

There is a considerable proportion of patients with chronic heart failure, especially DCM, which have auto-antibodies [21]. The high prevalence of viral genome in idiopathic forms [92] and the presence of myocardial auto-antibodies in asymptomatic relatives to familial DCM patients [20, 22] puts the heterogeneous clinical entity of DCM in an exceptional immunological position. The pathophysiological relevance of auto-antibodies in humans with heart failure has not yet been finally defined, whereas there is experimental evidence from rodent models that auto-antibodies especially against the β1-adrenergic receptor can induce a dilated cardiomyopathy phenotype [72]. The functional effect of β1-adrenergic receptors, which are of note also observed in up to 10 % of healthy control individuals, on intracellular β-adrenergic signaling in cardiomyocytes varies depending on their effect on receptor conformation and its internalization [12]. The binding sites for the pathogenic β1-adrenergic receptor antibodies are confined to portions of the extracellular loop domains where they induce agonist-like effects finally leading to apoptosis [29]. The presence of stimulating β1-adrenergic auto-antibodies was reported to independently predict the increased all-cause and cardiovascular mortality risk in a prospective study following patients with DCM over 10 years [129]. The prevalence of stimulating β1-adrenergic auto-antibodies was 26 % of patients with DCM in this cohort.

Several related studies have demonstrated that immuno-adsorption induces improvement of left ventricular function in patients with DCM [39, 125, 126]. It was also found that patients with auto-antibodies that induce cardio-depressant effects in vitro benefit most from this treatment [3, 128]. Immuno-adsorption with subsequent immunoglobulin substitution led to a reduction in the number of leukocytes and HLA class II expression in the myocardium of DCM patients, indicating that inflammatory activity within the myocardium is in fact reduced by treatment, whereas the extent of fibrosis was unchanged [127]. These promising results stimulated the design of a larger randomized trial on immune-adsorption which is currently underway (NCT00558584). As a different approach, there is another ongoing clinical trial evaluating a blocking peptide against stimulating β1-adrenergic auto-antibodies in DCM patients (COR-1 NCT01391507) [105].

### Immunoglobulins

A wide array of potential immuno-modulating effects has been attributed to intravenous immunoglobulins which demonstrated therapeutic efficacy in a variety of diseases with unrelated pathophysiology. However, clinical study results for treating heart failure are ambiguous. A small randomized trial in patients with symptomatic heart failure demonstrated a change in the balance between inflammatory and anti-inflammatory cytokines by immunoglobulin treatment. This effect was significantly correlated with an improvement in LVEF [52]. Another small randomized trial proved no effect of immunoglobulin treatment [101].

The underlying biological mechanism for the potential beneficial effects of immunoglobulins in DCM remains to be determined. A comprehensive overview of potential mechanism was recently provided by Gelfand [49]. Neutralization of auto-antibodies is one mechanism often discussed to contribute to the beneficial effect of immunoglobulins in auto-immune disease. However, a study in patients with DCM and ischemic cardiomyopathy found that intravenous immunoglobulins did not neutralize β1-adrenergic auto-antibodies [86]. Interpretation of the heterogenous study results is especially hampered by the fact that in most studies no comprehensive histological information from endomyocardial biopsies of all participants was available. Of note, there is a high rate of viral persistence in the myocardium of patients with idiopathic DCM [92]. Elimination of virus persistence was further demonstrated to improve the prognosis in a relatively small cohort of DCM patients [81]. Immunoglobulins have especially been shown to possess both anti-inflammatory and antiviral effects in experimental myocarditis [130]. This led to clinical evaluation of intravenous immunoglobulins for the treatment of viral cardiomyopathy. The most comprehensive current European Study on the Epidemiology and Treatment of Cardiac Inflammatory Disease (ESETCID) trial includes an elaborate treatment protocol treating cytomegalovirus, adeno-, and Parvo B19 positive inflammatory DCM patients with immunoglobulins [70, 95]. The final publication of the results of the trial is still pending.

Given the efficacy of immunoglobulins in attenuating virus replication in myocardium, it might thus be the case that the high prevalence of latent virus infection in the myocardium of DCM patients contributes to the positive effects of immunoglobulins in heterogeneous populations of DCM patients included in the above-mentioned trials.

### Bone marrow derived cells/progenitor cells

The field of therapeutic stem cell therapy has made great advance in the last years due to a number of clinical studies which are underway or just have been completed [58]. Most animal studies could not attribute the observed functional effects to regeneration via local engraftment and proliferation. Instead, paracrine effects of the transferred cells are generally believed to play a central role. Immuno-modulation by these cells might contribute to the overall beneficial effects of a variety of cell types. Especially for mesenchymal stem cells, manifold immuno-modulating properties have been described in this context [145]. Others have speculated that apoptotic cells, within the transferred cell preparations inducing immunosuppression, might play an important role [64, 90, 139]. This hypothesis would help explaining, why such a great variety of unrelated cell types produced an effect in experimental studies.

Most clinical trials were designed to improve repair/regeneration of the injured heart in the setting of myocardial infarction, while few studies yet addressed chronic heart failure patients.

The TOPCARE-CHD trial tested intracoronary treatment with either bone marrow cells or peripheral progenitor cells in a crossover design in patients with chronic ischemic heart failure. Transplantation of bone marrow cells was associated with moderate but significant improvement in the left ventricular ejection fraction after 3 months [7] and even improved survival in a registry-based analysis [6].

Another group reported similar promising results of intramyocardial delivery of bone marrow cells in chronic myocardial infarction and advanced chronic heart failure patients [111].

It was speculated that progenitor cell therapy might be more promising in non-ischemic heart failure patients, as the functional capacity of endogenous progenitor cells is more severely impaired in patients with ischemic cardiomyopathy than in DCM patients [137]. In the transplantation of progenitor cells and functional regeneration enhancement pilot trial in patients with non-ischemic dilated cardiomyopathy (TOPCARE-DCM) trial, autologous intracoronary bone marrow application resulted in significant improvement in LVEF at 1 year [41]. However, as it was often the case in progenitor cell trials, no adequate control group was studied in parallel in this pilot trial.

Recently, 5-year follow-up data of a well-designed, albeit not placebo-controlled and not double-blinded, randomized study investigating the long-term effects of intracoronary administration of G-CSF-mobilized CD34^+^ stem cells in patients with DCM were reported. During the follow-up period, cell therapy was associated with a moderate but significant improvement in cardiac function (mean increase of EF 5.7 %), coming along with an improved exercise capacity and even decreased mortality [148]. Interestingly, homing efficiency correlated with changes in EF indicating that transplanted cells elicited local effects in myocardium rather than inducing a systemic, e.g., immuno-modulating, effect.

However, none of the trials in chronic heart failure patients deeply assessed parameters of immune activation. Therefore, the contribution of putative immuno-modulatory effects of transferred cell preparations cannot be determined yet.

## New translational targets

There is a great variety of potential therapeutic targets in the field of pro-inflammatory signaling in heart failure. Perhaps, most experimental evidence has accumulated in experimental models of post-myocardial infarction heart failure concerning toll-like receptor and intracellular NFκB signaling (for review [42–44, 68]). However, the universal role of this system in innate immunity might induce unforeseeable side effects, especially concerning susceptibility to infection and auto-immunity. Thus, despite a good understanding of this signaling system, no therapeutic trial addressing chronic heart failure has been launched so far in this field.

Besides numerous signaling molecules like cytokines, chemokines, and their related receptors which have meanwhile been identified and targeted in animal studies two novel targets should be highlighted here.

### Complement

Complement is activated during acute and chronic cardiac injury in different animal models. Michael Carroll’s group demonstrated complement activation by ischemia/reperfusion in different organs by a single IgM clone that recognizes a neo-epitope on hypoxic cells [65, 159]. This clone is produced by a subset of B-lymphocytes called B-1 cells, the major source for natural IgM antibodies. Carroll’s group subsequently identified the relevant antigen as non-muscle myosin heavy chain II which becomes accessible to natural IgM after cell injury [159]. No experimental data are published on the role of natural IgM in chronic heart failure yet.

Glycoprotein-130 (gp130) is the common receptor of IL-6, which is elevated in patients with chronic heart failure [5]. Cardiomyocyte-specific gp130 mutant mice have a normal myocardial phenotype at baseline, but increased mortality and development of heart failure after experimental myocardial infarction. This was associated with increased expression of complement-activating mannose-binding lectin (MBL) [62]. This animal model suggested a link between IL-6 and chronic myocardial injury induced by complement activation.

MBL and IgMs are independent trigger mechanisms for initiating the complement cascade. Both pathways converge on the activation of complement component C3 into cleaved C3a. Mice deficient for either C3 or the membrane receptor for C3a (C3aR) is protected against chronic left ventricular remodeling after myocardial infarction (own unpublished results).

Accordingly, a prospective study in a chronic heart failure cohort comprising 182 patients demonstrated that complement activation is strongly linked to the outcome in chronic heart failure. Especially, high levels of activated C3, C3a, predicted cardiovascular events such as hospitalization and mortality [50]. However, studies in CHF patients are lacking yet. There are just clinical data for complement inhibition in the setting of acute ischemia. Whereas C1 esterase inhibitor treatment showed promising effects in surgically revascularized STEMI patients [38], anti-C5a therapy had no effect in STEMI patients [51].

Therefore, there is a considerable experimental evidence that at least after ischemic myocardial injury, there is an ongoing complement activity in myocardium. Interventions inhibiting complement activation, especially the central component C3/C3a may represent a novel target for prevention and treatment of chronic heart failure after large myocardial infarction.

### Regulatory T-cells

Most immuno-mechanisms studied in chronic heart failure till date address innate immunity, whereas adaptive immune mechanisms have gained much less interest so far. Recently, CD4^+^ regulatory T-cells came especially into focus.

**Fig. 2:**
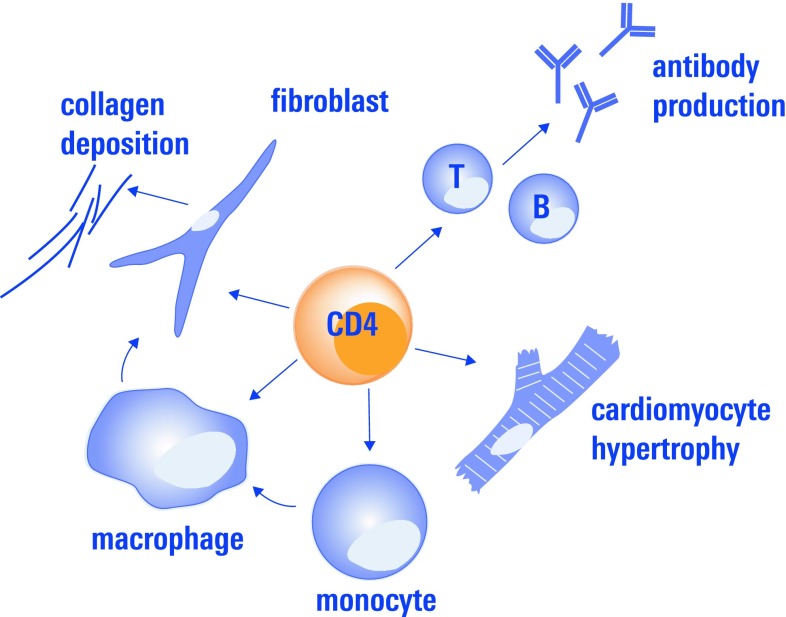
CD4^+^ regulatory T-cells modulate the function of T-effector cells and B-cells which leads to e.g., autoantibody production which has been found to contribute to cardiac dysfunction in several heart disease. Besides their classical regulatory role on adaptive immune response they have also been demonstrated to influence monocyte differentiation, which again influences fibrosis, and to attenuate myocardial hypertrophy

Regulatory CD4^+^ T-cells comprise a subset of thymus-derived T-cells which are characterized by intracellular expression of the transcription factor Foxp3 [115]. By means of cell surface staining for FACS analysis they are often defined as CD4^+^CD25^+^CD127^low^CD3^+^ T-cells. Regulatory T-cells are well known for preventing autoreactive immune responses which are associated with chronic myocardial diseases, such as auto-antibody formation [19]. As discussed below, there is further evidence from animal studies that they modulate fibrosis, cardiomyocyte hypertrophy, and monocyte activation in the myocardium, processes which constitute central mechanisms in myocardial remodeling (Fig. 2).

Regulatory CD4^+^ T-cells might mediate part of the clinical effect of immuno-modulating therapeutic principles, which was already tested in clinical studies: intravenous immunoglobulins beneficially modulate several unrelated auto-immune diseases. As discussed above, clinical trials indicate that they might also have beneficial effect in inflammatory cardiomyopathies. It was speculated that sustained peripheral expansion of antigen-specific CD4^+^CD25^+^Foxp3^+^ regulatory T-cells might contribute to their therapeutic effect [93].

Besides possible effects by neutralizing autoreactive antibodies, a recent study indicated that immuno-adsorption also modifies T cell-mediated immune reactions. Immuno-adsorption in patients with DCM was associated with a significant increase of regulatory T cells (CD4^+^CD25^+^CD127^low^), and a decrease of activated T cells (CD4^+^CD69^+^ and CD8^+^CD69^+^ cells and CD28^+^ T cells) [17]. The frequency of circulating regulatory T-cells also correlated with the hemodynamic response to immuno-adsorption [16].

Furthermore, reduced numbers and impaired suppressive function of CD4^+^CD25^+^ Foxp3^+^CD127^low^ cells were found in peripheral blood of patients with chronic heart failure [131]. There are also some experimental data indicating, that loss of functional regulatory T-cells is not just an epiphenomenon in chronic heart failure, but might also be relevant for myocardial remodeling in different disease states. We have recently demonstrated that myocardial infarction rapidly induces activation of proliferation of CD4^+^CD25^+^Foxp3^+^ T-cells in heart draining lymph nodes [66]. Our data indicate that the recognition of an antigen released from the injured myocardium is responsible for this activation process. Transfer of CD4^+^CD25^+^ T-cells was further reported to attenuate myocardial remodeling after experimental myocardial infarction in mice [97, 132]. Another group reported that transfer of CD4^+^CD25^+^ cells ameliorates cardiac hypertrophy and fibrosis in response to angiotensin II [84]. Of note, this was also associated with an amelioration of arrhythmogenic electric remodeling.

Therefore, therapeutic expansion and activation of regulatory T-cells might tackle several immunological mechanisms contributing to the heart failure syndrome: mitigation of the expression of pro-inflammatory cytokines, suppression of auto-antibody production, and modification of recruitment, and differentiation of monocytic heart infiltrating cells contributing to both tissue repair and remodeling (own unpublished data).

Besides transfer of autologous, ex vivo enriched regulatory T-cells, therapeutic expansion/activation in vivo might be more promising [31, 114]. Mucosal application of antigens is able to potently induce regulatory T-cells with specificity against it. Thus, mucosal administration of proteins induces secretion of anti-inflammatory cytokines, such as IL-10 at the anatomical site where the protein localizes. Frenkel et al. [46] have demonstrated that nasal application of troponin reduces infarct size after myocardial ischemia-reperfusion. This principle has not yet been tested in chronic heart failure, but might be a rather specific means of tuning immunity within the diseased myocardium.

Histone deacetylases (HDACs) catalyze the removal of acetyl groups from a variety of proteins. HDACs have been studied mainly in the context of chromatin, where they contribute to epigenetic regulation of gene expression by deacetylating nucleosomal histones. HDAC inhibitors stimulate T_reg_ production by promoting acetylation of the Foxp3 transcription factor, which is a key regulator of T_reg_ differentiation [149]. On the other hand, HDAC inhibitors beneficially affect myocardial remodeling, especially pathological hypertrophy in animal models [98]. The mechanism by which they do that is not quite clear, but induction of regulatory T-cells might significantly contribute to their effect. Therefore, this kind of drug might constitute a very interesting new therapeutic principle for targeting both inflammation and pathological fibrosis and hypertrophy in the context of chronic heart failure.

## Conclusion

Most completed trials on immuno-modulatory treatments were rather small including less than 100 patients/group. Data from randomized, placebo-controlled studies including more than 1,000 patients exist for TNF-α antagonists [25, 96]. The rosuvastatin trials, GISSI [134] and CORONA [77], as well as the ACCLAIM trial studying immuno-modulation by ex vivo modified autologous blood [147], also included more than 1,000 patients. All these chronic heart failure trials failed to demonstrate a benefit by meeting pre-specified endpoints. The sole large-scale randomized trial that was able to reveal some benefit was GISSI-HF which found a moderate, albeit significant benefit of ω-3 PUFA in addition to standard heart failure medication including a reduction in total mortality [133].

Why did so many clinical trials, which were all launched on the basis of promising experimental data, show no additional benefit to current standard heart failure therapy?

As one hypothetical explanation, it must be mentioned here that standard drugs addressing neuro-humoral activation, which are obligatory co-medication in current heart failure trials also might have relevant effects on inflammatory activation. Especially ACE inhibitors have immuno-modulatory properties, as components of the renin–angiotensin–aldosterone system are expressed on leukocytes. Especially, the angiotensin–aldosterone system in T-cells came recently into the focus. T-cells contain an endogenous renin–angiotensin system that modulates pro-inflammatory T-cell function [63]. Blocking angiotensin-converting enzymes on the other hand induces regulatory CD4^+^Foxp3^+^ T-cells which have the ability to dampen pathological inflammation and auto-immune by a variety of mechanisms [110]. Accordingly, it was reported that enalapril decreases IL-6 levels in patients with chronic systolic heart failure [53]. It remains unclear, however, whether this effect is due to a direct immuno-modulatory effect of the ACE inhibitor or rather a consequence of the improved overall hemodynamic and functional status under this medication. This relates to a central, yet unresolved question in the field, whether inflammation in heart failure is causative for or merely a consequence of heart failure.

One might ask whether there is still a justifiable rationale for conduction of future clinical trials targeting inflammation as adjunct to our current pharmacological heart failure therapy. In our opinion, there is good experimental evidence that inflammation causally contributes to myocardial dysfunction and, thus, still constitutes a reasonable and promising therapeutic target, but some questions have to be carefully addressed before designing a clinical trial.

First, which patients are best candidates for immuno-modulating treatment modalities? The hope to find a therapeutic principle that fits in all clinical scenarios of heart failure seems to be more and more unrealistic. It might be more promising in the future to individually characterize the immunologic status of a patient to decide whether he might benefit from the targeted immuno-modulation at the present time. Especially in patients with idiopathic DCM, this will have to include endomyocardial biopsies to gain more insights to define the present status of intramyocardial inflammatory activity and, especially, to exclude ongoing viral activity. Infectious (especially virus induced) cardiomyopathy might become accessible to a completely different treatment in the future. Immuno-adsorption in patients with auto-antibodies, which have been evaluated for their cardio-specificity and toxicity, exemplifies such a first individualized approach [128]. Complementary to the still underused endomyocardial biopsy-based analyses, which have inherent limitations mainly due to sampling errors, there is an urgent need for identifying novel biomarkers or imaging modalities to identify CHF patients who could benefit from immuno-modulation.

Monitoring of systemic, i.e., serum parameters of inflammation, especially cytokines, does likely not reflect myocardial inflammation, but is rather a marker of the overall clinical status. Therefore, one cannot conclude from the currently available serum parameters, which are often the only available biomaterial to monitor immunity in clinical studies, on the local effect on myocardial inflammation. Thus, at the moment, the real biological effect of immuno-modulating therapies, putatively beneficially influencing myocardial inflammation, cannot readily become assessed in clinical studies.

As a different approach, as opposed to CHF patients, the wound healing phase after an acute myocardial infarction, which is the most important cause for heart failure in the Western word, might constitute another attractive phase for preventive immuno-modulation [42]. Experimental animal studies indicate that there might be an early window for intervention to tune the immunological response to cardiac injury limiting infarct expansion and induction of ongoing remodeling in the remote myocardium.

Second, how can we better identify immuno-modulating treatment principles tested in animal models to be suited for further translational clinical evaluation? Here, pre-clinical testing of therapeutic modalities warrants more attention. Several treatment modalities went into clinical trials, mainly on the basis of promising effects in rodent models, indicating that immuno-modulation, as evidenced e.g., by cytokine expression profiling or genetic lack/gain of function studies, might be causative. Here, we have to pay more attention on the transferability of immunological phenomena from small animal models to man. This does not only relate to the estimation of the putative therapeutic impact, but also to potential side effects. In addition, before going into man for phase I/II trials, large-animal studies have to be advocated. However, even a very good understanding of the biological mechanism of an immuno-modulating therapeutic tested in different species does not prevent from unexpected effects in man which can, at best lead to lack of efficacy, but also to deleterious side effects [71].

Also essential, but widely underestimated, factors like age [11], co-medication, comorbidity, and last but not least, gender have to be drawn into consideration as much as possible in animal studies before thinking about clinical trials.

Therefore, we pledge for even greater effort spent on translational cardio-immunological research which goes beyond phenomenological description of beneficial effects on myocardial function and remodeling and its association with parameters of inflammation. Given the inherent limitation to monitor the systemic and, especially local immunological processes within the myocardium in patients, a deep understanding of the biological effects of a therapeutic principle in different experimental models is vital before starting clinical studies.

In addition, in this context, as many therapeutically principles have only been tested in the chronic myocardial infarction heart failure mouse model, other models of chronic heart failure, especially models of genetic and inflammatory cardiomyopathies, should receive more interest in the future.

Summing up the current evidence from both experimental and clinical studies discussed here, we would like to conclude that inflammation is a promising therapeutic target in the setting of CHF. Current state of evidence from clinical trials indicates that unspecific immuno-modulation, e.g., by steroids or immuno-adsorption, holds some promise especially in the “orphan” field of idiopathic/inflammatory, non-viral DCM (see Table 1). Thus, we can await even greater benefit from more targeted or even individualized treatment modalities which are eagerly awaited in the field as complementary therapeutic means to our current heart failure drugs.

**Table 1:** Randomized clinical studies evaluating immuno-modulating/anti-inflammatory treatments in CHF patients as discussed in the manuscript

Therapeutic intervention	Study	Patient number	Mean EF	NYHA status	Etiology	Main outcome
TNF-α binding						
Etanercept	[32]	18	23/29 %	III	Mixed	Improved functional parameters and EF
	[15]	47	16–21 %	III–IV	Mixed	
	[96]	2,048	22–24 %	III–IV	Mixed	Improved EF and remodeling parameters
Infliximab	[25]	150	23–25 %	III–IV	Mixed	Combined analysis of RECOVER and RENAISSANCE trials: no effect on clinical composite endpoint
						No clinical improvement, combined risk of death from any cause or hospitalization for heart failure increased in subgroup
Prednisone	[108]	102	18 %	nd	DCM	Transient improvement in EF
Prednisone + azathioprine	[150]	202	24 %	II–IV	DCM	Improved secondary endpoint of LV volume and EF
	[48]	85	27 %	II–IV	Virus-negative myocarditis	Improved EF and NYHA class
Thalidomide	[55]	56	25 %	II–III	Mixed	Improved EF
Rosuvastatin	[77]	5,011	31/33 %	II–IV	Mixed	No effect of primary combined endpoint
	[134]	4,631	33 %	II–IV	Mixed	No effect of primary combined endpoint
Pentoxifylline	[119]	39	24/25 %	II–III	DCM	Improved functional status after 6 months
	[120]	49	23 %	II–III	DCM	Improved functional class, and EF
	[122]	28	25/22 %	II–III	DCM	Improved EF and functional status
	[123]	18	13/16 %	IV	DCM	Improved EF
	[124]	38	23/27 %	II–III	ICM	Functional improvement
Isosorbide dinitrate + hydralazine	[135]	1,050	24 %	III–IV	Mixed	Improved composite endpoint in African–Americans. Study terminated prematurely for early benefit
Vitamin E	[74]	56	23 %	III–IV	Mixed	No clinical improvement, no effect on markers of oxidative stress after 12 weeks on treatment
Autologous modified blood	[144]	75	22 %	III–IV	Mixed	Reduced mortality and risk of hospitalization after 6 months
Polyunsaturated fatty acids	[133]	7,046	33 %	II–IV	Mixed	Improved composite endpoint
	[104]	43	24 %	III–IV	Non-ICM	Dose-dependent improved EF
Immuno-adsorption	[126]	22	28/27 %	III–IV	DCM	Improved EF, without control group
Immunoglobulins	[52]	40	26/28 %	II–III	Mixed	Improved EF
	[101]	62	25 %	I–IV	DCM, myocarditis	No change in EF
Intracoronary progenitor cells	[7]	92	43/39/41 %	I–III	ICM	Improved EF 3 months after infusion of bone marrow derived cells
	[111]	109	27 %	II–IV	ICM	Improved functional status and EF 12 months after infusion of bone marrow derived cells
	[148]	110	24/26 %	III	DCM	Improved functional status and EF after 5 years of infusion of CD34^+^ cells

If mean EF was not equal, EF for placebo/treatment groups were indicated

*nd* not determined, *ICM* ischemic cardiomyopathy, *DCM* dilative cardiomyopathy

## References

[1] GISSI Prevenzione Investigators (1999) Dietary supplementation with n-3 polyunsaturated fatty acids and vitamin E after myocardial infarction: results of the GISSI-Prevenzione trial. Gruppo Italiano per lo Studio della Sopravvivenza nell’Infarto miocardico. Lancet 354:447–455. pii:S0140673699070725 10465168

[2] Abel ED, Litwin SE, Sweeney G (2008). Cardiac remodeling in obesity. Physiol Rev.

[3] Ameling S, Herda LR, Hammer E, Steil L, Teumer A, Trimpert C, Dorr M, Kroemer HK, Klingel K, Kandolf R, Volker U, Felix SB (2012). Myocardial gene expression profiles and cardiodepressant autoantibodies predict response of patients with dilated cardiomyopathy to immunoadsorption therapy. Eur Heart J.

[4] Anker SD, Coats AJ (2002). How to RECOVER from RENAISSANCE? The significance of the results of RECOVER, RENAISSANCE, RENEWAL and ATTACH. Int J Cardiol.

[5] Askevold ET, Nymo S, Ueland T, Gravning J, Wergeland R, Kjekshus J, Yndestad A, Cleland JG, McMurray JJ, Aukrust P, Gullestad L (2013). Soluble glycoprotein 130 predicts fatal outcomes in chronic heart failure: analysis from the Controlled Rosuvastatin Multinational Trial in Heart Failure (CORONA). Circ Heart Fail.

[6] Assmus B, Fischer-Rasokat U, Honold J, Seeger FH, Fichtlscherer S, Tonn T, Seifried E, Schachinger V, Dimmeler S, Zeiher AM (2007). Transcoronary transplantation of functionally competent BMCs is associated with a decrease in natriuretic peptide serum levels and improved survival of patients with chronic postinfarction heart failure: results of the TOPCARE-CHD Registry. Circ Res.

[7] Assmus B, Honold J, Schachinger V, Britten MB, Fischer-Rasokat U, Lehmann R, Teupe C, Pistorius K, Martin H, Abolmaali ND, Tonn T, Dimmeler S, Zeiher AM (2006). Transcoronary transplantation of progenitor cells after myocardial infarction. N Engl J Med.

[8] Aukrust P, Ueland T, Lien E, Bendtzen K, Muller F, Andreassen AK, Nordoy I, Aass H, Espevik T, Simonsen S, Froland SS, Gullestad L (1999). Cytokine network in congestive heart failure secondary to ischemic or idiopathic dilated cardiomyopathy. Am J Cardiol.

[9] Bahrmann P, Hengst UM, Richartz BM, Figulla HR (2004). Pentoxifylline in ischemic, hypertensive and idiopathic-dilated cardiomyopathy: effects on left-ventricular function, inflammatory cytokines and symptoms. Eur J Heart Fail.

[10] Bianchi ME (2007). DAMPs, PAMPs and alarmins: all we need to know about danger. J Leukoc Biol.

[11] Boengler K, Schulz R, Heusch G (2009). Loss of cardioprotection with ageing. Cardiovasc Res.

[12] Bornholz B, Weidtkamp-Peters S, Schmitmeier S, Seidel CA, Herda LR, Felix SB, Lemoine H, Hescheler J, Nguemo F, Schafer C, Christensen MO, Mielke C, Boege F (2013). Impact of human autoantibodies on beta1-adrenergic receptor conformation, activity, and internalization. Cardiovasc Res.

[13] Borthwick LA, Wynn TA, Fisher AJ (2012). Cytokine mediated tissue fibrosis. Biochim Biophys Acta.

[14] Bozkurt B, Kribbs SB, Clubb FJ, Michael LH, Didenko VV, Hornsby PJ, Seta Y, Oral H, Spinale FG, Mann DL (1998). Pathophysiologically relevant concentrations of tumor necrosis factor-alpha promote progressive left ventricular dysfunction and remodeling in rats. Circulation.

[15] Bozkurt B, Torre-Amione G, Warren MS, Whitmore J, Soran OZ, Feldman AM, Mann DL (2001). Results of targeted anti-tumor necrosis factor therapy with etanercept (ENBREL) in patients with advanced heart failure. Circulation.

[16] Bulut D, Creutzenberg G, Mugge A (2012). The number of regulatory T cells correlates with hemodynamic improvement in patients with inflammatory dilated cardiomyopathy after immunoadsorption therapy. Scand J Immunol.

[17] Bulut D, Scheeler M, Wichmann T, Borgel J, Miebach T, Mugge A (2010). Effect of protein A immunoadsorption on T cell activation in patients with inflammatory dilated cardiomyopathy. Clin Res Cardiol.

[18] Busquets S, Aranda X, Ribas-Carbo M, Azcon-Bieto J, Lopez-Soriano FJ, Argiles JM (2003). Tumour necrosis factor-alpha uncouples respiration in isolated rat mitochondria. Cytokine.

[19] Bystry RS, Aluvihare V, Welch KA, Kallikourdis M, Betz AG (2001). B cells and professional APCs recruit regulatory T cells via CCL4. Nat Immunol.

[20] Caforio AL, Mahon NG, Baig MK, Tona F, Murphy RT, Elliott PM, McKenna WJ (2007). Prospective familial assessment in dilated cardiomyopathy: cardiac autoantibodies predict disease development in asymptomatic relatives. Circulation.

[21] Caforio AL, Mahon NG, McKenna WJ (2006). Clinical implications of anti-cardiac immunity in dilated cardiomyopathy. Ernst Schering Res Found Workshop.

[22] Caforio AL, Mahon NJ, Tona F, McKenna WJ (2002). Circulating cardiac autoantibodies in dilated cardiomyopathy and myocarditis: pathogenetic and clinical significance. Eur J Heart Fail.

[23] Canton M, Menazza S, Sheeran FL, Polverino de Laureto P, Di Lisa F, Pepe S (2011). Oxidation of myofibrillar proteins in human heart failure. J Am Coll Cardiol.

[24] Canton M, Skyschally A, Menabo R, Boengler K, Gres P, Schulz R, Haude M, Erbel R, Di Lisa F, Heusch G (2006). Oxidative modification of tropomyosin and myocardial dysfunction following coronary microembolization. Eur Heart J.

[25] Chung ES, Packer M, Lo KH, Fasanmade AA, Willerson JT (2003). Randomized, double-blind, placebo-controlled, pilot trial of infliximab, a chimeric monoclonal antibody to tumor necrosis factor-alpha, in patients with moderate-to-severe heart failure: results of the anti-TNF therapy against congestive heart failure (ATTACH) trial. Circulation.

[26] Cicoira M, Bolger AP, Doehner W, Rauchhaus M, Davos C, Sharma R, Al-Nasser FO, Coats AJ, Anker SD (2001). High tumour necrosis factor-alpha levels are associated with exercise intolerance and neurohormonal activation in chronic heart failure patients. Cytokine.

[27] Cooper LT (2009). Myocarditis. N Engl J Med.

[28] Corsten MF, Schroen B, Heymans S (2012). Inflammation in viral myocarditis: friend or foe?. Trends Mol Med.

[29] Dandel M, Wallukat G, Potapov E, Hetzer R (2012). Role of beta(1)-adrenoceptor autoantibodies in the pathogenesis of dilated cardiomyopathy. Immunobiology.

[30] Dangas G, Konstadoulakis MM, Epstein SE, Stefanadis CI, Kymionis GD, Toutouza MG, Liakos C, Sadaniantz A, Cohen AM, Chesebro JH, Toutouzas PK (2000). Prevalence of autoantibodies against contractile proteins in coronary artery disease and their clinical implications. Am J Cardiol.

[31] Daniele N, Scerpa MC, Landi F, Caniglia M, Miele MJ, Locatelli F, Isacchi G, Zinno F (2011). T(reg) cells: collection, processing, storage and clinical use. Pathol Res Pract.

[32] Deswal A, Bozkurt B, Seta Y, Parilti-Eiswirth S, Hayes FA, Blosch C, Mann DL (1999). Safety and efficacy of a soluble P75 tumor necrosis factor receptor (Enbrel, etanercept) in patients with advanced heart failure. Circulation.

[33] Deswal A, Petersen NJ, Feldman AM, Young JB, White BG, Mann DL (2001). Cytokines and cytokine receptors in advanced heart failure: an analysis of the cytokine database from the Vesnarinone trial (VEST). Circulation.

[34] Dinh W, Futh R, Nickl W, Krahn T, Ellinghaus P, Scheffold T, Bansemir L, Bufe A, Barroso MC, Lankisch M (2009). Elevated plasma levels of TNF-alpha and interleukin-6 in patients with diastolic dysfunction and glucose metabolism disorders. Cardiovasc Diabetol.

[35] Dorge H, Schulz R, Belosjorow S, Post H, van de Sand A, Konietzka I, Frede S, Hartung T, Vinten-Johansen J, Youker KA, Entman ML, Erbel R, Heusch G (2002). Coronary microembolization: the role of TNF-alpha in contractile dysfunction. J Mol Cell Cardiol.

[36] Fairweather D, Kaya Z, Shellam GR, Lawson CM, Rose NR (2001). From infection to autoimmunity. J Autoimmun.

[37] Falcao-Pires I, Palladini G, Goncalves N, van der Velden J, Moreira-Goncalves D, Miranda-Silva D, Salinaro F, Paulus WJ, Niessen HW, Perlini S, Leite-Moreira AF (2011). Distinct mechanisms for diastolic dysfunction in diabetes mellitus and chronic pressure-overload. Basic Res Cardiol.

[38] Fattouch K, Bianco G, Speziale G, Sampognaro R, Lavalle C, Guccione F, Dioguardi P, Ruvolo G (2007). Beneficial effects of C1 esterase inhibitor in ST-elevation myocardial infarction in patients who underwent surgical reperfusion: a randomised double-blind study. Eur J Cardiothorac Surg.

[39] Felix SB, Staudt A, Landsberger M, Grosse Y, Stangl V, Spielhagen T, Wallukat G, Wernecke KD, Baumann G, Stangl K (2002). Removal of cardiodepressant antibodies in dilated cardiomyopathy by immunoadsorption. J Am Coll Cardiol.

[40] Ferdinand KC (2007). African American heart failure trial: role of endothelial dysfunction and heart failure in African Americans. Am J Cardiol.

[41] Fischer-Rasokat U, Assmus B, Seeger FH, Honold J, Leistner D, Fichtlscherer S, Schachinger V, Tonn T, Martin H, Dimmeler S, Zeiher AM (2009). A pilot trial to assess potential effects of selective intracoronary bone marrow-derived progenitor cell infusion in patients with nonischemic dilated cardiomyopathy: final 1-year results of the transplantation of progenitor cells and functional regeneration enhancement pilot trial in patients with nonischemic dilated cardiomyopathy. Circ Heart Fail.

[42] Frantz S, Bauersachs J, Ertl G (2009). Post-infarct remodelling: contribution of wound healing and inflammation. Cardiovasc Res.

[43] Frantz S, Ertl G, Bauersachs J (2007). Mechanisms of disease: toll-like receptors in cardiovascular disease. Nat Clin Pract Cardiovasc Med.

[44] Frantz S, Ertl G, Bauersachs J (2008). Toll-like receptor signaling in the ischemic heart. Front Biosci.

[45] Frantz S, Hu K, Bayer B, Gerondakis S, Strotmann J, Adamek A, Ertl G, Bauersachs J (2006). Absence of NF-kappaB subunit p50 improves heart failure after myocardial infarction. FASEB J.

[46] Frenkel D, Pachori AS, Zhang L, Dembinsky-Vaknin A, Farfara D, Petrovic-Stojkovic S, Dzau VJ, Weiner HL (2009). Nasal vaccination with troponin reduces troponin specific T-cell responses and improves heart function in myocardial ischemia-reperfusion injury. Int Immunol.

[47] Friedrichs GS, Swillo RE, Jow B, Bridal T, Numann R, Warner LM, Killar LM, Sidek K (2002). Sphingosine modulates myocyte electrophysiology, induces negative inotropy, and decreases survival after myocardial ischemia. J Cardiovasc Pharmacol.

[48] Frustaci A, Russo MA, Chimenti C (2009). Randomized study on the efficacy of immunosuppressive therapy in patients with virus-negative inflammatory cardiomyopathy: the TIMIC study. Eur Heart J.

[49] Gelfand EW (2012). Intravenous immune globulin in autoimmune and inflammatory diseases. N Engl J Med.

[50] Gombos T, Forhecz Z, Pozsonyi Z, Szeplaki G, Kunde J, Fust G, Janoskuti L, Karadi I, Prohaszka Z (2012). Complement anaphylatoxin C3a as a novel independent prognostic marker in heart failure. Clin Res Cardiol.

[51] Granger CB, Mahaffey KW, Weaver WD, Theroux P, Hochman JS, Filloon TG, Rollins S, Todaro TG, Nicolau JC, Ruzyllo W, Armstrong PW (2003). Pexelizumab, an anti-C5 complement antibody, as adjunctive therapy to primary percutaneous coronary intervention in acute myocardial infarction: the COMplement inhibition in Myocardial infarction treated with Angioplasty (COMMA) trial. Circulation.

[52] Gullestad L, Aass H, Fjeld JG, Wikeby L, Andreassen AK, Ihlen H, Simonsen S, Kjekshus J, Nitter-Hauge S, Ueland T, Lien E, Froland SS, Aukrust P (2001). Immunomodulating therapy with intravenous immunoglobulin in patients with chronic heart failure. Circulation.

[53] Gullestad L, Aukrust P, Ueland T, Espevik T, Yee G, Vagelos R, Froland SS, Fowler M (1999). Effect of high- versus low-dose angiotensin converting enzyme inhibition on cytokine levels in chronic heart failure. J Am Coll Cardiol.

[54] Gullestad L, Semb AG, Holt E, Skardal R, Ueland T, Yndestad A, Froland SS, Aukrust P (2002). Effect of thalidomide in patients with chronic heart failure. Am Heart J.

[55] Gullestad L, Ueland T, Fjeld JG, Holt E, Gundersen T, Breivik K, Folling M, Hodt A, Skardal R, Kjekshus J, Andreassen A, Kjekshus E, Wergeland R, Yndestad A, Froland SS, Semb AG, Aukrust P (2005). Effect of thalidomide on cardiac remodeling in chronic heart failure: results of a double-blind, placebo-controlled study. Circulation.

[56] Gullestad L, Ueland T, Vinge LE, Finsen A, Yndestad A, Aukrust P (2012). Inflammatory cytokines in heart failure: mediators and markers. Cardiology.

[57] Hare JM, Mangal B, Brown J, Fisher C, Freudenberger R, Colucci WS, Mann DL, Liu P, Givertz MM, Schwarz RP (2008). Impact of oxypurinol in patients with symptomatic heart failure. Results of the OPT–CHF study. J Am Coll Cardiol.

[58] Heusch G (2011). SCIPIO brings new momentum to cardiac cell therapy. Lancet.

[59] Heusch G, Schulz R (2011). A radical view on the contractile machinery in human heart failure. J Am Coll Cardiol.

[60] Heusch P, Canton M, Aker S, van de Sand A, Konietzka I, Rassaf T, Menazza S, Brodde OE, Di Lisa F, Heusch G, Schulz R (2010). The contribution of reactive oxygen species and p38 mitogen-activated protein kinase to myofilament oxidation and progression of heart failure in rabbits. Br J Pharmacol.

[61] Heymans S, Hirsch E, Anker SD, Aukrust P, Balligand JL, Cohen-Tervaert JW, Drexler H, Filippatos G, Felix SB, Gullestad L, Hilfiker-Kleiner D, Janssens S, Latini R, Neubauer G, Paulus WJ, Pieske B, Ponikowski P, Schroen B, Schultheiss HP, Tschope C, Van Bilsen M, Zannad F, McMurray J, Shah AM (2009). Inflammation as a therapeutic target in heart failure? A scientific statement from the Translational Research Committee of the Heart Failure Association of the European Society of Cardiology. Eur J Heart Fail.

[62] Hilfiker-Kleiner D, Shukla P, Klein G, Schaefer A, Stapel B, Hoch M, Muller W, Scherr M, Theilmeier G, Ernst M, Hilfiker A, Drexler H (2010). Continuous glycoprotein-130-mediated signal transducer and activator of transcription-3 activation promotes inflammation, left ventricular rupture, and adverse outcome in subacute myocardial infarction. Circulation.

[63] Hoch NE, Guzik TJ, Chen W, Deans T, Maalouf SA, Gratze P, Weyand C, Harrison DG (2009). Regulation of T-cell function by endogenously produced angiotensin II. Am J Physiol Regul Integr Comp Physiol.

[64] Hoetzenecker K, Assinger A, Lichtenauer M, Mildner M, Schweiger T, Starlinger P, Jakab A, Berenyi E, Pavo N, Zimmermann M, Gabriel C, Plass C, Gyongyosi M, Volf I, Ankersmit HJ (2012). Secretome of apoptotic peripheral blood cells (APOSEC) attenuates microvascular obstruction in a porcine closed chest reperfused acute myocardial infarction model: role of platelet aggregation and vasodilation. Basic Res Cardiol.

[65] Hofmann U, Bauersachs J, Frantz S (2010). Nothing but natural: targeting natural IgM in ischaemia/reperfusion injury. Cardiovasc Res.

[66] Hofmann U, Beyersdorf N, Weirather J, Podolskaya A, Bauersachs J, Ertl G, Kerkau T, Frantz S (2012). Activation of CD4+ T lymphocytes improves wound healing and survival after experimental myocardial infarction in mice. Circulation.

[67] Hofmann U, Domeier E, Frantz S, Laser M, Weckler B, Kuhlencordt P, Heuer S, Keweloh B, Ertl G, Bonz AW (2003). Increased myocardial oxygen consumption by TNF-alpha is mediated by a sphingosine signaling pathway. Am J Physiol Heart Circ Physiol.

[68] Hofmann U, Ertl G, Frantz S (2011). Toll-like receptors as potential therapeutic targets in cardiac dysfunction. Expert Opin Ther Targets.

[69] Hori M, Nishida K (2009). Oxidative stress and left ventricular remodelling after myocardial infarction. Cardiovasc Res.

[70] Hufnagel G, Pankuweit S, Richter A, Schonian U, Maisch B (2000). The European Study of Epidemiology and Treatment of Cardiac Inflammatory Diseases (ESETCID). First epidemiological results. Herz.

[71] Hunig T (2012). The storm has cleared: lessons from the CD28 superagonist TGN1412 trial. Nat Rev Immunol.

[72] Jahns R, Boivin V, Hein L, Triebel S, Angermann CE, Ertl G, Lohse MJ (2004). Direct evidence for a beta 1-adrenergic receptor-directed autoimmune attack as a cause of idiopathic dilated cardiomyopathy. J Clin Investig.

[73] Keith M, Geranmayegan A, Sole MJ, Kurian R, Robinson A, Omran AS, Jeejeebhoy KN (1998). Increased oxidative stress in patients with congestive heart failure. J Am Coll Cardiol.

[74] Keith ME, Jeejeebhoy KN, Langer A, Kurian R, Barr A, O’Kelly B, Sole MJ (2001). A controlled clinical trial of vitamin E supplementation in patients with congestive heart failure. Am J Clin Nutr.

[75] Kell R, Haunstetter A, Dengler TJ, Zugck C, Kubler W, Haass M (2002). Do cytokines enable risk stratification to be improved in NYHA functional class III patients? Comparison with other potential predictors of prognosis. Eur Heart J.

[76] Kindermann I, Kindermann M, Kandolf R, Klingel K, Bultmann B, Muller T, Lindinger A, Bohm M (2008). Predictors of outcome in patients with suspected myocarditis. Circulation.

[77] Kjekshus J, Apetrei E, Barrios V, Bohm M, Cleland JG, Cornel JH, Dunselman P, Fonseca C, Goudev A, Grande P, Gullestad L, Hjalmarson A, Hradec J, Janosi A, Kamensky G, Komajda M, Korewicki J, Kuusi T, Mach F, Mareev V, McMurray JJ, Ranjith N, Schaufelberger M, Vanhaecke J, van Veldhuisen DJ, Waagstein F, Wedel H, Wikstrand J (2007). Rosuvastatin in older patients with systolic heart failure. N Engl J Med.

[78] Kleinbongard P, Heusch G, Schulz R (2010). TNFalpha in atherosclerosis, myocardial ischemia/reperfusion and heart failure. Pharmacol Ther.

[79] Kubota T, McTiernan CF, Frye CS, Slawson SE, Lemster BH, Koretsky AP, Demetris AJ, Feldman AM (1997). Dilated cardiomyopathy in transgenic mice with cardiac-specific overexpression of tumor necrosis factor-alpha. Circ Res.

[80] Kuhl U, Pauschinger M, Noutsias M, Seeberg B, Bock T, Lassner D, Poller W, Kandolf R, Schultheiss HP (2005). High prevalence of viral genomes and multiple viral infections in the myocardium of adults with “idiopathic” left ventricular dysfunction. Circulation.

[81] Kuhl U, Pauschinger M, Schwimmbeck PL, Seeberg B, Lober C, Noutsias M, Poller W, Schultheiss HP (2003). Interferon-beta treatment eliminates cardiotropic viruses and improves left ventricular function in patients with myocardial persistence of viral genomes and left ventricular dysfunction. Circulation.

[82] Kumar A, Paladugu B, Mensing J, Parrillo JE (2007). Nitric oxide-dependent and -independent mechanisms are involved in TNF-alpha-induced depression of cardiac myocyte contractility. Am J Physiol Regul Integr Comp Physiol.

[83] Kuwahara F, Kai H, Tokuda K, Takeya M, Takeshita A, Egashira K, Imaizumi T (2004). Hypertensive myocardial fibrosis and diastolic dysfunction: another model of inflammation?. Hypertension.

[84] Kvakan H, Luft FC, Muller DN (2009). Role of the immune system in hypertensive target organ damage. Trends Cardiovasc Med.

[85] Kwak B, Mulhaupt F, Myit S, Mach F (2000). Statins as a newly recognized type of immunomodulator. Nat Med.

[86] Larsson L, Mobini R, Aukrust P, Gullestad L, Wallukat G, Waagstein F, Fu M (2004). Beneficial effect on cardiac function by intravenous immunoglobulin treatment in patients with dilated cardiomyopathy is not due to neutralization of anti-receptor autoantibody. Autoimmunity.

[87] Lavie CJ, Milani RV, Mehra MR, Ventura HO (2009). Omega-3 polyunsaturated fatty acids and cardiovascular diseases. J Am Coll Cardiol.

[88] LeGal YM, Morrissey LL (1990). Methylprednisolone interventions in myocardial infarction: a controversial subject. Can J Cardiol.

[89] Li YY, Feng YQ, Kadokami T, McTiernan CF, Draviam R, Watkins SC, Feldman AM (2000). Myocardial extracellular matrix remodeling in transgenic mice overexpressing tumor necrosis factor alpha can be modulated by anti-tumor necrosis factor alpha therapy. Proc Natl Acad Sci USA.

[90] Lichtenauer M, Mildner M, Hoetzenecker K, Zimmermann M, Podesser BK, Sipos W, Berenyi E, Dworschak M, Tschachler E, Gyongyosi M, Ankersmit HJ (2011). Secretome of apoptotic peripheral blood cells (APOSEC) confers cytoprotection to cardiomyocytes and inhibits tissue remodelling after acute myocardial infarction: a preclinical study. Basic Res Cardiol.

[91] Lotze U, Egerer R, Gluck B, Zell R, Sigusch H, Erhardt C, Heim A, Kandolf R, Bock T, Wutzler P, Figulla HR (2010). Low level myocardial parvovirus B19 persistence is a frequent finding in patients with heart disease but unrelated to ongoing myocardial injury. J Med Virol.

[92] Lotze U, Egerer R, Tresselt C, Gluck B, Dannberg G, Stelzner A, Figulla HR (2004). Frequent detection of parvovirus B19 genome in the myocardium of adult patients with idiopathic dilated cardiomyopathy. Med Microbiol Immunol.

[93] Maddur MS, Othy S, Hegde P, Vani J, Lacroix-Desmazes S, Bayry J, Kaveri SV (2010). Immunomodulation by intravenous immunoglobulin: role of regulatory T cells. J Clin Immunol.

[94] Mahrholdt H, Wagner A, Deluigi CC, Kispert E, Hager S, Meinhardt G, Vogelsberg H, Fritz P, Dippon J, Bock CT, Klingel K, Kandolf R, Sechtem U (2006). Presentation, patterns of myocardial damage, and clinical course of viral myocarditis. Circulation.

[95] Maisch B, Hufnagel G, Kolsch S, Funck R, Richter A, Rupp H, Herzum M, Pankuweit S (2004). Treatment of inflammatory dilated cardiomyopathy and (peri)myocarditis with immunosuppression and i.v. immunoglobulins. Herz.

[96] Mann DL, McMurray JJ, Packer M, Swedberg K, Borer JS, Colucci WS, Djian J, Drexler H, Feldman A, Kober L, Krum H, Liu P, Nieminen M, Tavazzi L, van Veldhuisen DJ, Waldenstrom A, Warren M, Westheim A, Zannad F, Fleming T (2004). Targeted anticytokine therapy in patients with chronic heart failure: results of the Randomized Etanercept Worldwide Evaluation (RENEWAL). Circulation.

[97] Matsumoto K, Ogawa M, Suzuki J, Hirata Y, Nagai R, Isobe M (2011). Regulatory T lymphocytes attenuate myocardial infarction-induced ventricular remodeling in mice. Int Heart J.

[98] McKinsey TA (2011). Targeting inflammation in heart failure with histone deacetylase inhibitors. Mol Med.

[99] McMurray JJ, Adamopoulos S, Anker SD, Auricchio A, Bohm M, Dickstein K, Falk V, Filippatos G, Fonseca C, Gomez-Sanchez MA, Jaarsma T, Kober L, Lip GY, Maggioni AP, Parkhomenko A, Pieske BM, Popescu BA, Ronnevik PK, Rutten FH, Schwitter J, Seferovic P, Stepinska J, Trindade PT, Voors AA, Zannad F, Zeiher A (2012). ESC Guidelines for the diagnosis and treatment of acute and chronic heart failure 2012: The Task Force for the Diagnosis and Treatment of Acute and Chronic Heart Failure 2012 of the European Society of Cardiology. Developed in collaboration with the Heart Failure Association (HFA) of the ESC. Eur Heart J.

[100] McMurray JJ, Adamopoulos S, Anker SD, Auricchio A, Bohm M, Dickstein K, Falk V, Filippatos G, Fonseca C, Gomez-Sanchez MA, Jaarsma T, Kober L, Lip GY, Maggioni AP, Parkhomenko A, Pieske BM, Popescu BA, Ronnevik PK, Rutten FH, Schwitter J, Seferovic P, Stepinska J, Trindade PT, Voors AA, Zannad F, Zeiher A, Bax JJ, Baumgartner H, Ceconi C, Dean V, Deaton C, Fagard R, Funck-Brentano C, Hasdai D, Hoes A, Kirchhof P, Knuuti J, Kolh P, McDonagh T, Moulin C, Reiner Z, Sechtem U, Sirnes PA, Tendera M, Torbicki A, Vahanian A, Windecker S, Bonet LA, Avraamides P, Ben Lamin HA, Brignole M, Coca A, Cowburn P, Dargie H, Elliott P, Flachskampf FA, Guida GF, Hardman S, Iung B, Merkely B, Mueller C, Nanas JN, Nielsen OW, Orn S, Parissis JT, Ponikowski P (2012). ESC guidelines for the diagnosis and treatment of acute and chronic heart failure 2012: the Task Force for the Diagnosis and Treatment of Acute and Chronic Heart Failure 2012 of the European Society of Cardiology. Developed in collaboration with the Heart Failure Association (HFA) of the ESC. Eur J Heart Fail.

[101] McNamara DM, Holubkov R, Starling RC, Dec GW, Loh E, Torre-Amione G, Gass A, Janosko K, Tokarczyk T, Kessler P, Mann DL, Feldman AM (2001). Controlled trial of intravenous immune globulin in recent-onset dilated cardiomyopathy. Circulation.

[102] Meldrum DR (1998). Tumor necrosis factor in the heart. Am J Physiol.

[103] Miles EA, Calder PC (2012). Influence of marine n-3 polyunsaturated fatty acids on immune function and a systematic review of their effects on clinical outcomes in rheumatoid arthritis. Br J Nutr.

[104] Moertl D, Hammer A, Steiner S, Hutuleac R, Vonbank K, Berger R (2011). Dose-dependent effects of omega-3-polyunsaturated fatty acids on systolic left ventricular function, endothelial function, and markers of inflammation in chronic heart failure of nonischemic origin: a double-blind, placebo-controlled, 3-arm study. Am Heart J.

[105] Munch G, Boivin-Jahns V, Holthoff HP, Adler K, Lappo M, Truol S, Degen H, Steiger N, Lohse MJ, Jahns R, Ungerer M (2012). Administration of the cyclic peptide COR-1 in humans (phase I study): ex vivo measurements of anti-beta1-adrenergic receptor antibody neutralization and of immune parameters. Eur J Heart Fail.

[106] Nolan Y, Minogue A, Vereker E, Bolton AE, Campbell VA, Lynch MA (2002). Attenuation of LPS-induced changes in synaptic activity in rat hippocampus by Vasogen’s Immune Modulation Therapy. Neuroimmunomodulation.

[107] Parissis JT, Venetsanou KF, Mentzikof DG, Ziras NG, Kefalas CG, Karas SM (1999). Tumor necrosis factor-alpha serum activity during treatment of acute decompensation of cachectic and non-cachectic patients with advanced congestive heart failure. Scand Cardiovasc J.

[108] Parrillo JE, Cunnion RE, Epstein SE, Parker MM, Suffredini AF, Brenner M, Schaer GL, Palmeri ST, Cannon RO, Alling D (1989). A prospective, randomized, controlled trial of prednisone for dilated cardiomyopathy. N Eng J Med.

[109] Pei H, Qu Y, Lu X, Yu Q, Lian K, Liu P, Yan W, Liu J, Ma Y, Liu Y, Li C, Li W, Lau WB, Zhang H, Tao L (2013). Cardiac-derived adiponectin induced by long-term insulin treatment ameliorates myocardial ischemia/reperfusion injury in type 1 diabetic mice via AMPK signaling. Basic Res Cardiol.

[110] Platten M, Youssef S, Hur EM, Ho PP, Han MH, Lanz TV, Phillips LK, Goldstein MJ, Bhat R, Raine CS, Sobel RA, Steinman L (2009). Blocking angiotensin-converting enzyme induces potent regulatory T cells and modulates TH1- and TH17-mediated autoimmunity. Proc Natl Acad Sci USA.

[111] Pokushalov E, Romanov A, Chernyavsky A, Larionov P, Terekhov I, Artyomenko S, Poveshenko O, Kliver E, Shirokova N, Karaskov A, Dib N (2010). Efficiency of intramyocardial injections of autologous bone marrow mononuclear cells in patients with ischemic heart failure: a randomized study. J Cardiovasc Transl Res.

[112] Rauchhaus M, Doehner W, Francis DP, Davos C, Kemp M, Liebenthal C, Niebauer J, Hooper J, Volk HD, Coats AJ, Anker SD (2000). Plasma cytokine parameters and mortality in patients with chronic heart failure. Circulation.

[113] Richardson P, McKenna W, Bristow M, Maisch B, Mautner B, O’Connell J, Olsen E, Thiene G, Goodwin J, Gyarfas I, Martin I, Nordet P (1996). Report of the 1995 World Health Organization/International Society and Federation of Cardiology Task Force on the Definition and Classification of cardiomyopathies. Circulation.

[114] Safinia N, Sagoo P, Lechler R, Lombardi G (2010). Adoptive regulatory T cell therapy: challenges in clinical transplantation. Curr Opin Organ Transpl.

[115] Sakaguchi S, Miyara M, Costantino CM, Hafler DA (2010). FOXP3+ regulatory T cells in the human immune system. Nat Rev Immunol.

[116] Shaw SM, Shah MK, Williams SG, Fildes JE (2009). Immunological mechanisms of pentoxifylline in chronic heart failure. Eur J Heart Fail.

[117] Shibata R, Ouchi N, Ito M, Kihara S, Shiojima I, Pimentel DR, Kumada M, Sato K, Schiekofer S, Ohashi K, Funahashi T, Colucci WS, Walsh K (2004). Adiponectin-mediated modulation of hypertrophic signals in the heart. Nat Med.

[118] Singer G, Granger DN (2007). Inflammatory responses underlying the microvascular dysfunction associated with obesity and insulin resistance. Microcirculation.

[119] Skudicky D, Bergemann A, Sliwa K, Candy G, Sareli P (2001). Beneficial effects of pentoxifylline in patients with idiopathic dilated cardiomyopathy treated with angiotensin-converting enzyme inhibitors and carvedilol: results of a randomized study. Circulation.

[120] Skudicky D, Sliwa K, Bergemann A, Candy G, Sareli P (2000). Reduction in Fas/APO-1 plasma concentrations correlates with improvement in left ventricular function in patients with idiopathic dilated cardiomyopathy treated with pentoxifylline. Heart.

[121] Sliwa K, Skudicky D, Candy G, Bergemann A, Hopley M, Sareli P (2002). The addition of pentoxifylline to conventional therapy improves outcome in patients with peripartum cardiomyopathy. Eur J Heart Fail.

[122] Sliwa K, Skudicky D, Candy G, Wisenbaugh T, Sareli P (1998). Randomised investigation of effects of pentoxifylline on left-ventricular performance in idiopathic dilated cardiomyopathy. Lancet.

[123] Sliwa K, Woodiwiss A, Candy G, Badenhorst D, Libhaber C, Norton G, Skudicky D, Sareli P (2002). Effects of pentoxifylline on cytokine profiles and left ventricular performance in patients with decompensated congestive heart failure secondary to idiopathic dilated cardiomyopathy. Am J Cardiol.

[124] Sliwa K, Woodiwiss A, Kone VN, Candy G, Badenhorst D, Norton G, Zambakides C, Peters F, Essop R (2004). Therapy of ischemic cardiomyopathy with the immunomodulating agent pentoxifylline: results of a randomized study. Circulation.

[125] Staudt A, Bohm M, Knebel F, Grosse Y, Bischoff C, Hummel A, Dahm JB, Borges A, Jochmann N, Wernecke KD, Wallukat G, Baumann G, Felix SB (2002). Potential role of autoantibodies belonging to the immunoglobulin G-3 subclass in cardiac dysfunction among patients with dilated cardiomyopathy. Circulation.

[126] Staudt A, Hummel A, Ruppert J, Dorr M, Trimpert C, Birkenmeier K, Krieg T, Staudt Y, Felix SB (2006). Immunoadsorption in dilated cardiomyopathy: 6-month results from a randomized study. Am Heart J.

[127] Staudt A, Schaper F, Stangl V, Plagemann A, Bohm M, Merkel K, Wallukat G, Wernecke KD, Stangl K, Baumann G, Felix SB (2001). Immunohistological changes in dilated cardiomyopathy induced by immunoadsorption therapy and subsequent immunoglobulin substitution. Circulation.

[128] Staudt A, Staudt Y, Dorr M, Bohm M, Knebel F, Hummel A, Wunderle L, Tiburcy M, Wernecke KD, Baumann G, Felix SB (2004). Potential role of humoral immunity in cardiac dysfunction of patients suffering from dilated cardiomyopathy. J Am Coll Cardiol.

[129] Stork S, Boivin V, Horf R, Hein L, Lohse MJ, Angermann CE, Jahns R (2006). Stimulating autoantibodies directed against the cardiac beta1-adrenergic receptor predict increased mortality in idiopathic cardiomyopathy. Am Heart J.

[130] Takada H, Kishimoto C, Hiraoka Y (1995). Therapy with immunoglobulin suppresses myocarditis in a murine coxsackievirus B3 model. Antiviral and anti-inflammatory effects. Circulation.

[131] Tang TT, Ding YJ, Liao YH, Yu X, Xiao H, Xie JJ, Yuan J, Zhou ZH, Liao MY, Yao R, Cheng Y, Cheng X (2010). Defective circulating CD4CD25+Foxp3+CD127(low) regulatory T-cells in patients with chronic heart failure. Cell Physiol Biochem.

[132] Tang TT, Yuan J, Zhu ZF, Zhang WC, Xiao H, Xia N, Yan XX, Nie SF, Liu J, Zhou SF, Li JJ, Yao R, Liao MY, Tu X, Liao YH, Cheng X (2012). Regulatory T cells ameliorate cardiac remodeling after myocardial infarction. Basic Res Cardiol.

[133] Tavazzi L, Maggioni AP, Marchioli R, Barlera S, Franzosi MG, Latini R, Lucci D, Nicolosi GL, Porcu M, Tognoni G (2008). Effect of n-3 polyunsaturated fatty acids in patients with chronic heart failure (the GISSI-HF trial): a randomised, double-blind, placebo-controlled trial. Lancet.

[134] Tavazzi L, Maggioni AP, Marchioli R, Barlera S, Franzosi MG, Latini R, Lucci D, Nicolosi GL, Porcu M, Tognoni G (2008). Effect of rosuvastatin in patients with chronic heart failure (the GISSI-HF trial): a randomised, double-blind, placebo-controlled trial. Lancet.

[135] Taylor AL, Ziesche S, Yancy C, Carson P, D’Agostino R, Ferdinand K, Taylor M, Adams K, Sabolinski M, Worcel M, Cohn JN (2004). Combination of isosorbide dinitrate and hydralazine in blacks with heart failure. N Engl J Med.

[136] Taylor AL, Ziesche S, Yancy CW, Carson P, Ferdinand K, Taylor M, Adams K, Olukotun AY, Ofili E, Tam SW, Sabolinski ML, Worcel M, Cohn JN (2007). Early and sustained benefit on event-free survival and heart failure hospitalization from fixed-dose combination of isosorbide dinitrate/hydralazine: consistency across subgroups in the African–American Heart Failure Trial. Circulation.

[137] Theiss HD, David R, Engelmann MG, Barth A, Schotten K, Naebauer M, Reichart B, Steinbeck G, Franz WM (2007). Circulation of CD34+ progenitor cell populations in patients with idiopathic dilated and ischaemic cardiomyopathy (DCM and ICM). Eur Heart J.

[138] Thielmann M, Dorge H, Martin C, Belosjorow S, Schwanke U, van De Sand A, Konietzka I, Buchert A, Kruger A, Schulz R, Heusch G (2002). Myocardial dysfunction with coronary microembolization: signal transduction through a sequence of nitric oxide, tumor necrosis factor-alpha, and sphingosine. Circ Res.

[139] Thum T, Bauersachs J, Poole-Wilson PA, Volk HD, Anker SD (2005). The dying stem cell hypothesis: immune modulation as a novel mechanism for progenitor cell therapy in cardiac muscle. J Am Coll Cardiol.

[140] Tinkel J, Hassanain H, Khouri SJ (2012). Cardiovascular antioxidant therapy: a review of supplements, pharmacotherapies, and mechanisms. Cardiol Rev.

[141] Torre-Amione G, Anker SD, Bourge RC, Colucci WS, Greenberg BH, Hildebrandt P, Keren A, Motro M, Moye LA, Otterstad JE, Pratt CM, Ponikowski P, Rouleau JL, Sestier F, Winkelmann BR, Young JB (2008). Results of a non-specific immunomodulation therapy in chronic heart failure (ACCLAIM trial): a placebo-controlled randomised trial. Lancet.

[142] Torre-Amione G, Kapadia S, Lee J, Bies RD, Lebovitz R, Mann DL (1995). Expression and functional significance of tumor necrosis factor receptors in human myocardium. Circulation.

[143] Torre-Amione G, Kapadia S, Lee J, Durand JB, Bies RD, Young JB, Mann DL (1996). Tumor necrosis factor-alpha and tumor necrosis factor receptors in the failing human heart. Circulation.

[144] Torre-Amione G, Sestier F, Radovancevic B, Young J (2004). Effects of a novel immune modulation therapy in patients with advanced chronic heart failure: results of a randomized, controlled, phase II trial. J Am Coll Cardiol.

[145] van den Akker F, Deddens JC, Doevendans PA, Sluijter JP (2012). Cardiac stem cell therapy to modulate inflammation upon myocardial infarction. Biochim Biophys Acta.

[146] Vanderheyden M, Paulus WJ, Voss M, Knuefermann P, Sivasubramanian N, Mann D, Baumgarten G (2005). Myocardial cytokine gene expression is higher in aortic stenosis than in idiopathic dilated cardiomyopathy. Heart.

[147] Voll RE, Herrmann M, Roth EA, Stach C, Kalden JR, Girkontaite I (1997). Immunosuppressive effects of apoptotic cells. Nature.

[148] Vrtovec B, Poglajen G, Lezaic L, Sever M, Domanovic D, Cernelc P, Socan A, Schrepfer S, Torre-Amione G, Haddad F, Wu JC (2012). Effects of Intracoronary Cd34 + Stem Cell Transplantation in Non-Ischemic Dilated Cardiomyopathy Patients: 5-Year Follow Up. Circ Res.

[149] Wang L, de Zoeten EF, Greene MI, Hancock WW (2009). Immunomodulatory effects of deacetylase inhibitors: therapeutic targeting of FOXP3 + regulatory T cells. Nat Rev Drug Discov.

[150] Wojnicz R, Nowalany-Kozielska E, Wojciechowska C, Glanowska G, Wilczewski P, Niklewski T, Zembala M, Polonski L, Rozek MM, Wodniecki J (2001). Randomized, placebo-controlled study for immunosuppressive treatment of inflammatory dilated cardiomyopathy: two-year follow-up results. Circulation.

[151] Wynn TA, Barron L (2010). Macrophages: master regulators of inflammation and fibrosis. Semin Liver Dis.

[152] Yamagishi K, Iso H, Date C, Fukui M, Wakai K, Kikuchi S, Inaba Y, Tanabe N, Tamakoshi A (2008). Fish, omega-3 polyunsaturated fatty acids, and mortality from cardiovascular diseases in a nationwide community-based cohort of Japanese men and women the JACC (Japan Collaborative Cohort Study for Evaluation of Cancer Risk) Study. J Am Coll Cardiol.

[153] Yamaguchi O, Higuchi Y, Hirotani S, Kashiwase K, Nakayama H, Hikoso S, Takeda T, Watanabe T, Asahi M, Taniike M, Matsumura Y, Tsujimoto I, Hongo K, Kusakari Y, Kurihara S, Nishida K, Ichijo H, Hori M, Otsu K (2003). Targeted deletion of apoptosis signal-regulating kinase 1 attenuates left ventricular remodeling. Proc Natl Acad Sci USA.

[154] Yokoyama T, Nakano M, Bednarczyk JL, McIntyre BW, Entman M, Mann DL (1997). Tumor necrosis factor-alpha provokes a hypertrophic growth response in adult cardiac myocytes. Circulation.

[155] Yokoyama T, Vaca L, Rossen RD, Durante W, Hazarika P, Mann DL (1993). Cellular basis for the negative inotropic effects of tumor necrosis factor-alpha in the adult mammalian heart. J Clin Invest.

[156] Yu Q, Horak K, Larson DF (2006). Role of T lymphocytes in hypertension-induced cardiac extracellular matrix remodeling. Hypertension.

[157] Yu Q, Vazquez R, Khojeini EV, Patel C, Venkataramani R, Larson DF (2009). IL-18 induction of osteopontin mediates cardiac fibrosis and diastolic dysfunction in mice. Am J Physiol Heart Circ Physiol.

[158] Zhang H, Potter BJ, Cao JM, Zhang C (2011). Interferon-gamma induced adipose tissue inflammation is linked to endothelial dysfunction in type 2 diabetic mice. Basic Res Cardiol.

[159] Zhang M, Alicot EM, Chiu I, Li J, Verna N, Vorup-Jensen T, Kessler B, Shimaoka M, Chan R, Friend D, Mahmood U, Weissleder R, Moore FD, Carroll MC (2006). Identification of the target self-antigens in reperfusion injury. J Exp Med.

[160] Zhu J, Liu M, Kennedy RH, Liu SJ (2006). TNF-alpha-induced impairment of mitochondrial integrity and apoptosis mediated by caspase-8 in adult ventricular myocytes. Cytokine.

